# Impact of visit‐to‐visit fasting plasma glucose variability on the development of diabetes: The mediation by insulin resistance

**DOI:** 10.1111/1753-0407.13253

**Published:** 2022-02-16

**Authors:** Jiji Xu, Ling Li, Shuangqing Huang, Haihong Song, Jinli Gao, Hengru Ni, Hong Lin, Shuangyuan Wang, Mian Li, Tiange Wang, Zhiyun Zhao, Min Xu, Jieli Lu, Yufang Bi, Yu Xu, Xiaozhong Qian

**Affiliations:** ^1^ Songnan Town Community Health Service Center Shanghai China; ^2^ Department of Endocrine and Metabolic Diseases, Shanghai Institute of Endocrine and Metabolic Diseases, Ruijin Hospital Shanghai Jiaotong University School of Medicine Shanghai China; ^3^ Shanghai National Clinical Research Center for Metabolic Diseases, Key Laboratory for Endocrine and Metabolic Diseases of the National Health Commission of the PR China, Shanghai Key Laboratory for Endocrine Tumor, State Key Laboratory of Medical Genomics, Ruijin Hospital Shanghai Jiaotong University School of Medicine Shanghai China; ^4^ Department of Pharmacy, Ruijin Hospital Shanghai Jiaotong University School of Medicine Shanghai China

**Keywords:** diabetes, fasting plasma glucose, mediation analysis, visit‐to‐visit variability, 就诊间变异性, 空腹血糖, 糖尿病, 中介分析。

## Abstract

**Background:**

Little is known about the risk of diabetes due to higher glycemic variability and the underlying mechanisms. We aimed to examine the association of visit‐to‐visit variability (VVV) in fasting plasma glucose (FPG) with incident diabetes in Chinese adults and whether the association was mediated by changes in insulin resistance (IR).

**Methods:**

We included 1856 community residents without a history of diabetes and having attended 3 examinations in 2008, 2009, and 2013 respectively. The SD, the average successive variability (ASV), the coefficient of variation (CV), and the variability independent of the mean (VIM) of three recorded FPG measurements were calculated for each participant, and SD, ASV, CV, and VIM were used as a measure of VVV in FPG. Incident diabetes was defined according to the 1999 World Health Organization criteria. IR was evaluated using the homeostatic model assessment (HOMA).

**Results:**

A total of 153 (8.2%) participants developed incident diabetes at the third visit. Compared with the lowest tertile (0–5.83 mg/dl) of FPG‐SD, the highest tertile (9.55–74.17 mg/dl) was associated with a 148% increased risk of diabetes (odds ratio [OR], 2.48; 95% confidence interval [CI], 1.36–4.49), after adjustment for covariates including mean FPG at 3 visits. Mediation analyses suggested that changes in IR (ΔHOMA‐IR) might mediate 17.3% of the association between increased FPG‐SD and elevated diabetes risk. Similar results were found for FPG‐CV, FPG‐ASV, and FPG‐VIM.

**Conclusions:**

The VVV in FPG was significantly associated with risks of diabetes in Chinese adults, which was partially mediated by changes in IR.

## Graphical abstract

**Highlights**

- A greater long‐term variability in fasting plasma glucose is significantly associated with an increased risk of incident diabetes.
- The association between fasting plasma glucose variability and incident diabetes could be partially mediated by an elevated homeostatic model assessment of insulin resistance.
- Fasting plasma glucose variability should be considered in early prevention of diabetes.

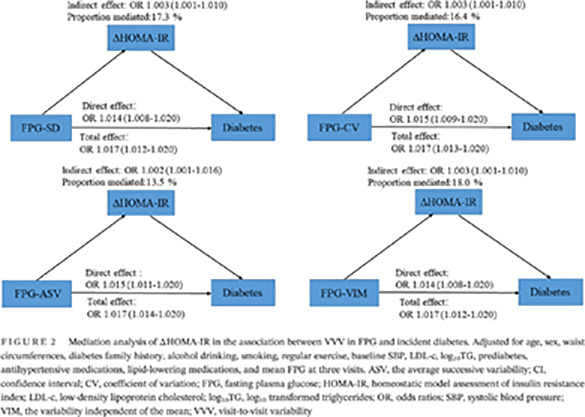

## 摘要

**背景:**

由于较高的血糖变异性和潜在机制，人们对糖尿病的风险知之甚少。我们旨在研究中国成年人空腹血糖 (FPG) 的就诊间变异 (VVV) 与糖尿病发病的关系，以及这种关联是否由胰岛素抵抗 (IR)介导。

**方法:**

我们纳入了1856名无糖尿病病史的社区居民，分别于2008年, 2009年和2013年参加了3次检查。为每个参与者计算了3次FPG测量值的标准差 (SD), 平均连续变异性 (ASV), 变异系数 (CV) 和与平均值无关的变异性 (VIM) 。SD, ASV, CV和 VIM 被用作 FPG 中 VVV 的量度。根据 1999 年世界卫生组织标准定义糖尿病。使用稳态模型评估 (HOMA) 评估 IR。

**结果:**

共有 153 名 (8.2%) 参与者在第 3 次就诊时出现糖尿病。校正包括 3 次就诊时的平均 FPG在内的协变量后，与 FPG‐SD 最低三分位数 (0 ‐ 5.83 mg/dL) 相比，最高三分位数 (9.55‐74.17 mg/dL) 糖尿病风险增加 148% (OR = 2.48, 95% CI 1.36‐4.49)。中介分析表明，IR的变化(ΔHOMA‐IR)可能介导 FPG‐SD 增加与糖尿病风险升高之间 17.3% 的关联。对于 FPG‐CV , FPG‐ASV 和 FPG‐VIM 也发现了类似的结果。

**结论:**

FPG 中的 VVV 与中国成年人患糖尿病的风险显着相关，这部分是由 IR 的变化介导的。

## 1 INTRODUCTION

Diabetes is an important cause of cardiovascular disease morbidity and mortality worldwide and poses a great disease burden on healthcare system.^1^, ^2^ Current management of diabetes uses glycated hemoglobin A1c (HbA1c), blood glucose, or glycemic variability to monitor glucose control.^3^ Glycemic variability is an assessment of fluctuations in glycemia, which includes short‐term (within‐day or between‐days) and long‐term glycemic variability. The latter refers to fluctuations within several months or years and is usually assessed by visit‐to‐visit variability (VVV) of HbA1c or fasting plasma glucose (FPG).^3^, ^4^, ^5^, ^6^, ^7^

Recent observational studies suggest that the visit‐to‐visit glycemic variability might be an independent risk factor for incident diabetes.^8^, ^9^ For example, a nationwide population‐based cohort study in Korea found that increased variability of FPG is associated with a higher risk for the development of diabetes independent of mean FPG level.^8^ However, another study in Denmark showed that higher variability of HbA1c was not significantly associated with incident type 2 diabetes.^10^ Therefore, current evidence of the impact of visit‐to‐visit glycemic variability on the development of diabetes is controversial.

Besides, these studies did not examine the underlying mechanisms linking glycemic variability and incident diabetes. Basic laboratory studies have demonstrated that higher plasma glucose variability had a greater effect on oxidative stress and endothelial function compared with a sustained hyperglycemia.^11^, ^12^ Furthermore, the oxidative stress‐activated signaling pathway leads to insulin resistance (IR), which is the main pathogenic mechanism of diabetes.^13^ Therefore, it is possible that glycemic variability may facilitate the development of diabetes via altered insulin sensitivity. However, no data are available on the potential role of IR as a mediator in the association between glycemic variability and incident diabetes.

The purpose of this study was to investigate (a) the association of VVV of FPG with risks of diabetes and (b) the mediating effect of changes in IR on this potential association using a population cohort of community residents who have participated in several metabolic health examinations.

## 2 METHODS

### 2.1 Study population

The present analysis was performed based on an ongoing cohort of Chinese adults in Shanghai. The details of the cohort have been described previously.^14^ Briefly, participants underwent three examination visits. At the first visit (June and July 2008), 10 185 adults aged ≥40 years from Songnan Community participated in the screening examination. All the subjects completed a brief survey including FPG, blood pressure (BP), and lipid levels evaluation. In the second visit (June through August 2009), participants were randomly selected from registered permanent residents of the first visit in a ratio of 1.0 diabetes (with FPG 126 mg/dl or higher or with a history of diabetes) to 1.2 impaired glucose regulation (with FPG 100 to 125 mg/dl and without a history of diabetes) to 1.44 normal glucose regulation (with FPG less than 100 mg/dl and without a history of diabetes). All the selected participants completed a detailed survey including a detailed questionnaire, physical examinations, a standard 75 g oral glucose tolerance test (OGTT), and blood and urine sampling. In the third visit (between March and May 2013), individuals who participated in the previous two visits were invited to have reevaluations of FPG, OGTT 2 h plasma glucose (OGTT 2 h‐PG), and fasting serum insulin. A total of 2883 adults completed all three visits.

**FIGURE 1:**
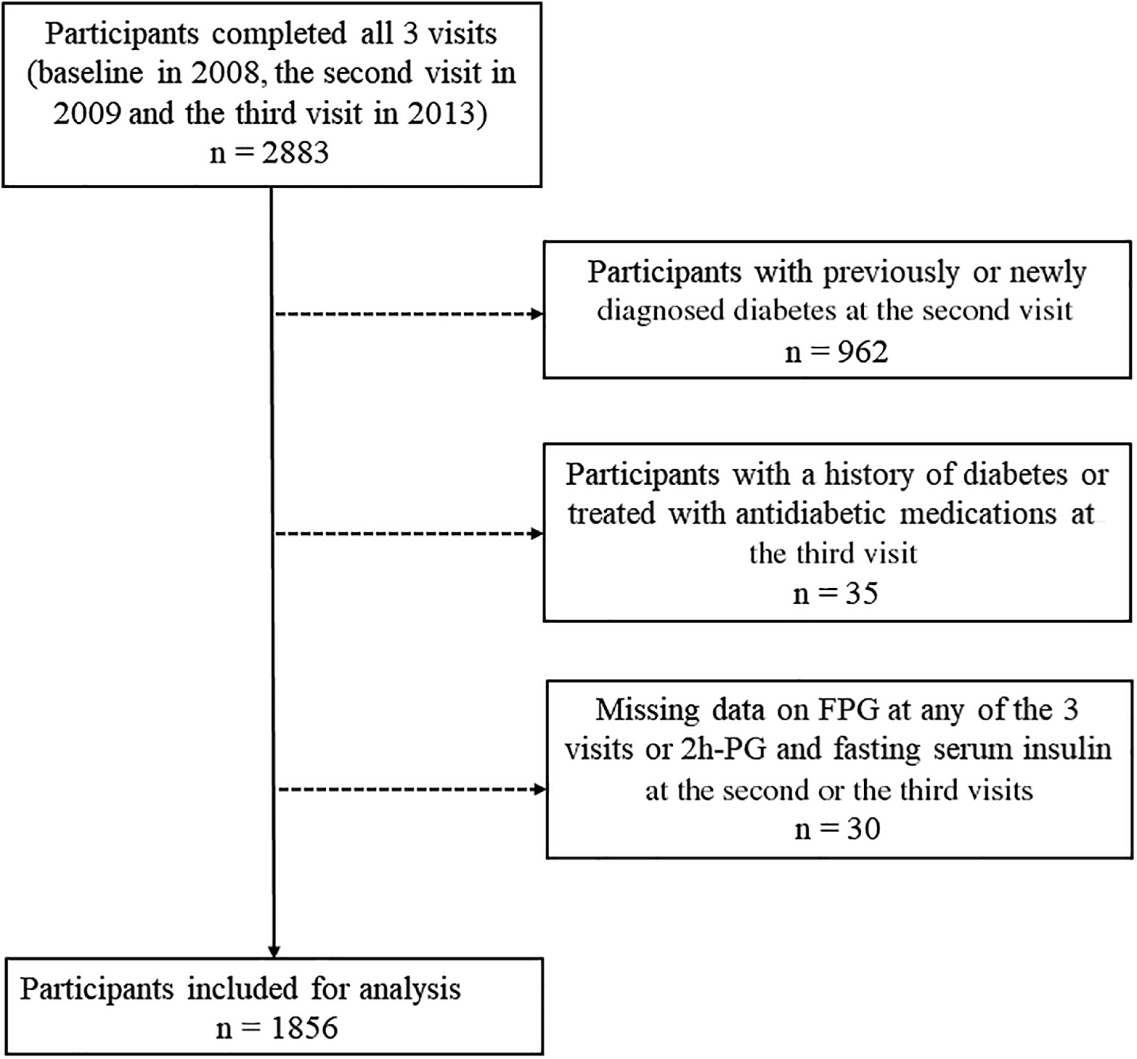
Flow chart of the study population. FPG, fasting plasma glucose

For the current analysis, participants with previously or newly diagnosed diabetes at the second visit (*n* = 962), with a history of diabetes or treated with antidiabetic medications at the third visit (*n* = 35), or with missing data on FPG at any of the three visits or OGTT 2 h‐PG and fasting serum insulin at the second or the third visits (*n* = 30) were excluded. Therefore, 1856 individuals were eventually included in the current analysis (Figure 1).

The study protocol was approved by the Committee on Human Research at Ruijin Hospital, Shanghai Jiao Tong University School of Medicine. All participants provided written informed consent.

### 2.2 Data collection

Data were collected from local community clinics at the first, the second, and the third visits.

Detailed information regarding participant demographics, medical history, family history, and lifestyle factors (eg, smoking and drinking status) was obtained by a standard questionnaire. The International Physical Activity Questionnaire was used to assess physical activity.^15^ Questions including frequency and duration of mild, moderate, and vigorous activities in the previous week were asked. Regular exercise was defined as ≥150 min/week moderate‐intensity activity, ≥75 min/week vigorous‐intensity activity, or ≥150 min/week moderate‐ and vigorous‐intensity activity.^16^ Body mass index (BMI) was calculated as body weight (kg) divided by the square of body height in meters (kg/m^2^). Three seated BP measurements were made with 1 min intervals after a 5 min rest, using an automated electronic device (OMRON Model HEM‐752, Omron, Dalian, China). The mean value of three measurements was used for analysis.

After an overnight fast of more than 10 h, venous blood samples were collected at all three visits. At the first visit, FPG of all participants was measured. At the second and the third visits, all participants underwent a standard 75 g OGTT after an overnight fast. Blood samples were obtained at 0 h and 2 h during the test. Laboratory tests measured FPG (all three visits), OGTT 2 h‐PG (the second and the third visits), fasting serum insulin (the second and the third visits), and lipid profiles (all three visits). Plasma glucose was measured using the glucose oxidase method on an automated analyzer (ADVIA‐1650 Chemistry System, Bayer Corporation, Leverkusen, Germany). Serum insulin was measured by electrochemiluminescence assay (Roche Diagnostics, Basel, Switzerland). Lipid profile including triglycerides (TG), total cholesterol (TC), high‐density lipoprotein cholesterol (HDL‐c), and low‐density lipoprotein cholesterol (LDL‐c) was measured by an automatic analyzer (ADVIA‐1650 Chemistry System, Bayer Corporation, Leverkusen, Germany).

### 2.3 Measures of glycemic variability

Long‐term visit‐to‐visit FPG variability was evaluated using four variability measures from all three visits, calculating the following for individual participants:(1) the SD, (2) the average successive variability (ASV), (3) the coefficient of variation (CV), and (4) the variability independent of the mean (VIM). ASV is the average of absolute difference between successive values of FPG.VIM was calculated as SD/mean^β^, where β is the regression coefficient based on ln of FPG‐SD over ln of mean.^4^, ^6^, ^8^, ^17^

### 2.4 Definitions

Incident diabetes was defined according to the 1999 World Health Organization criteria as FPG ≥126 mg/dl (7.0 mmol/L) or OGTT 2 h‐PG ≥200 mg/dl (11.1 mmol/L) or a self‐reported previous diagnosis of diabetes by healthcare professionals.^18^, ^19^ Homeostatic model assessment of IR index (HOMA‐IR) was calculated as follows: fasting insulin (μIU/ml) × fasting glucose (mg/dl)/405.^20^ Hypertension was defined as systolic blood pressure (SBP) ≥140 mm Hg or diastolic blood pressure (DBP) ≥90 mm Hg or taking antihypertensive medications. Dyslipidemia was defined as taking lipid‐lowering medications, TC ≥240 mg/dl, TG ≥200 mg/dl, HDL‐c < 40 mg/dl, or LDL‐c ≥ 160 mg/dl.^21^

### 2.5 Statistical analysis

Continuous variables are shown as the means ± SDs or medians (interquartile ranges). Categorical variables are presented as numbers (percentages). Differences between participants were compared by one‐way ANOVA for continuous variables or χ^2^ test for categorical variables. Skewed variables such as TG, fasting serum insulin, HOMA‐IR, and ΔHOMA‐IR (HOMA‐IR at the third visit minus HOMA‐IR at the second visit) were log_10_‐transformed before analysis.

We used multivariable logistic regression models to evaluate the associations of each measure of FPG variability (SD, CV, ASV, and VIM) with incident diabetes. This was done modeling each measure of FPG variability as a continuous variable as well as tertiles of each measure of FPG variability with the lowest tertile serving as the reference. Three models were used. Model 1 was adjusted for age, sex, waist circumference, diabetes family history, current drinking, current smoking, and regular exercise; model 2 was additionally adjusted for baseline SBP, LDL‐c, log_10_TG, prediabetes, antihypertensive medications, lipid‐lowering medications, and mean FPG at three visits; and model 3 was additionally adjusted for ΔHOMA‐IR. *p* values for trend across tertiles of FPG variability (SD, CV, ASV, and VIM) were calculated in regression analyses using the SD, CV, ASV, and VIM tertiles as an ordinal variable. Adjusted odds ratios (OR) and corresponding 95% confidence intervals (CIs) were calculated. We also conducted stratified analyses of associations between FPG variability and incident diabetes among participants with increased or decreased HOMA‐IR between the second and the third visits.

In order to explore whether ΔHOMA‐IR affected the association between VVV in FPG and risk of incident diabetes, we further conducted causal mediation analysis. A mediator should be linked to both the exposure and the outcome. The association between ΔHOMA‐IR and VVV in FPG was evaluated using generalized linear regression models (Proc genmod) and the regression coefficient (β) and 95% CI were calculated for associations of FPG variability (SD, CV, ASV, and VIM) tertiles and ΔHOMA‐IR. The association between ΔHOMA‐IR and incident diabetes was evaluated using logistic regression models.

R package was used to conduct the mediation analysis of ΔHOMA‐IR on the association between VVV in FPG and risk of incident diabetes. Logistic regression models using the ternary VVV in FPG variable as the exposure, ΔHOMA‐IR as the mediator, and diabetes as the outcome were examined. A linear regression model using the ternary VVV in FPG variable as the exposure and ΔHOMA‐IR as outcome was also examined. These two types of regression models were integrated to obtain the effect mediated by ΔHOMA‐IR in relation to the FPG variability‐diabetes association. All models were adjusted for age, sex, waist circumference, diabetes family history, current drinking, current smoking, regular exercise, baseline SBP, LDL‐c, log_10_TG, and mean FPG at 3 visits.

Significance tests were two tailed, with a *p* value <0.05 considered as statistically significant. All statistical analyses were performed using SAS version 9.4 (SAS Institute Inc, Cary, NC) and R software (version 3.6.1; R Foundation for Statistical Computing).

## 3 RESULTS

### 3.1 Characteristics of study participants

The FPG levels of study participants fluctuated during the follow‐up (Supplementary Figure S1 in Appendix S1). The characteristics of the participants grouped according to the tertiles of the FPG variability (FPG‐SD) are listed in Table 1. Overall, the mean age of the study population was 57.8 ± 9.1 years and 35.9% (*n* = 666) of participants were men. A total of 8.2% (*n* = 153) of participants developed diabetes. Participants in higher tertiles of the FPG‐SD were more likely to have significantly higher levels of BMI, waist circumference, BP, fasting serum insulin, HOMA‐IR, ΔHOMA‐IR, and FPG (all *p* < .05). The proportions of hypertension, dyslipidemia, and antihypertensive medications were significantly higher in those with higher tertiles of the FPG‐SD (all *p* < .05). Similar findings were observed when three other FPG variability measurements (FPG‐CV, FPG‐ASV, and FPG‐VIM) were used (Supplementary Tables S1–S3 in Appendix S1).

**TABLE 1:** Characteristics of participants according to the tertiles of the FPG variability (FPG‐SD)

	T1 (0‐5.83 mg/dl)	T2 (5.83‐9.55 mg/dl)	T3 (9.55‐74.17 mg/dl)	*P* value
Participants, *n*	620	618	618	
Age (years)	57.5 ± 8.8	57.8 ± 9.7	58.0 ± 8.8	.595
Men, *n* (%)	211 (34.0)	214 (34.6)	241 (39.0)	.139
Family history of diabetes, *n* (%)	85 (13.7)	86 (13.9)	87 (14.1)	.985
BMI (kg/m^2^)	24.6 ± 3.3	24.8 ± 3.7	25.2 ± 3.6	.014
Waist circumference (cm)	82.6 ± 9.0	83.3 ± 9.8	84.1 ± 9.3	.022
Lifestyle factors, *n* (%)				
Current smoking	149 (24.0)	142 (23.0)	147 (23.8)	.901
Current drinking	97 (15.7)	107 (17.3)	113 (18.3)	.459
Regular exercise	34 (5.5)	29 (4.7)	35 (5.7)	.719
Blood pressure (mm Hg)				
SBP	129 ± 21	129 ± 20	133 ± 20	<.001
DBP	79 ± 10	78 ± 10	81 ± 10	<.001
Lipid profile (mg/dl)				
TG	100.8 (71.4‐141.0)	98.1 (72.2‐144.4)	105.3 (72.9‐152.6)	.156
TC	196.0 ± 32.9	197.3 ± 33.9	197.0 ± 36.5	.745
HDL‐c	55.0 ± 11.0	54.8 ± 11.6	54.1 ± 12.1	.379
LDL‐c	93.9 ± 25.1	95.3 ± 24.2	93.8 ± 25.0	.478
Fasting serum insulin, at the second visit (μIU/ml)	6.3 (4.2‐8.9)	6.2 (4.2‐9.3)	7.0 (4.6‐10.4)	.001
Fasting serum insulin, at the third visit (μIU/ml)	5.8 (4.4‐7.8)	6.0 (4.3‐8.2)	7.4 (5.4‐10.1)	<.001
HOMA‐IR at the second visit	1.4 (0.9‐2.0)	1.4 (0.9‐2.1)	1.6 (0.99‐2.4)	.001
HOMA‐IR at the third visit	1.3 (1.0‐1.8)	1.5 (1.0‐2.0)	2.0 (1.4‐2.8)	<.001
Δ HOMA‐IR	−0.02 (−0.39‐0.30)	0.11 (−0.42‐0.45)	0.43 (−0.12‐0.94)	<.001
FPG at baseline (mg/dl)	88.4 ± 7.4	87.2 ± 7.7	89.5 ± 10.1	<.001
FPG variability				
SD (mg/dl)	3.9 ± 1.4	7.7 ± 1.1	14.1 ± 5.7	<.001
CV (%)	4.4 ± 1.5	8.4 ± 1.3	14.5 ± 4.7	<.001
ASV (mg/dl)	4.7 ± 2.0	8.7 ± 2.4	15.8 ± 6.9	<.001
VIM (%)	0.84 ± 0.29	1.6 ± 0.23	2.9 ± 1.1	<.001
Hypertension, *n* (%)	221 (35.7)	215 (34.8)	284 (46.0)	<.001
Dyslipidemia, *n* (%)	141 (22.7)	147 (23.8)	180 (29.1)	.021
Medications				
Antihypertensive medications at any of the 3 visits, *n* (%)	152 (24.5)	139 (22.5)	209 (33.8)	<.001
Lipid‐lowering medications at any of the 3 visits, *n* (%)	3 (0.48)	6 (0.97)	7 (1.1)	.438

*Note*: Data are means ± SDs or medians (IQRs) for continuous variables, or numbers (percentages) for categorical variables.

*Note*: There were 1 missing value for family history of diabetes, 1 missing value for BMI, 2 missing values for SBP, 2 missing values for DBP, and 1 missing value for hypertension, respectively.

Abbreviations: ASV, the average successive variability; BMI, body mass index; CV, coefficient of variation; DBP, diastolic blood pressure; FPG, fasting plasma glucose; HDL‐c, high‐density lipoprotein cholesterol; HOMA‐IR, homeostatic model assessment of insulin resistance index; IQR, interquartile range; LDL‐c, low‐density lipoprotein cholesterol; SBP, systolic blood pressure; TC, total cholesterol; TG, triglyceride; VIM, variability independent of the mean.

### 3.2 Association of VVV in FPG with incident diabetes

Table 2 shows the associations of glycemic variability with risks of incident diabetes. After adjustment for confounding factors including mean FPG at three visits, the risk of diabetes development increased with increasing SD tertiles of FPG (*p* value for trend <.001). When compared with the lowest tertile (0–5.83 mg/dl), participants in the highest tertile (9.55–74.17 mg/dl) of FPG‐SD had a 148% increased risk for diabetes (OR = 2.48; 95% CI, 1.36–4.49). In addition, the risk increased by 14% for each unit increment in FPG‐SD (OR = , 1.14; 95% CI, 1.09–1.19). Similar results were observed with other measures of VVV in FPG including FPG‐CV, FPG‐ASV, and FPG‐VIM.

**TABLE 2:** Odds ratios and 95% confidence intervals of diabetes risks in association with the VVV in FPG

	Incident cases/No. of participants	Cumulative incidence (%)	OR (95% CI)		
			Model 1	Model 2	Model 3
Tertiles of SD (mg/dl)					
T1 (0–5.83)	17/620	2.7	1(ref.)	1(ref.)	1(ref.)
T2 (5.83–9.55)	26/618	4.2	1.49 (0.80–2.78)	1.36 (0.70–2.64)	1.29 (0.67–2.49)
T3 (9.55–74.17)	110/618	17.8	7.45 (4.40–12.63)	3.21 (1.80–5.72)	2.48 (1.36–4.49)
*P* for trend		<.001	<.001	<.001	.001
Each 1 increment			1.24 (1.20–1.28)	1.16 (1.11–1.20)	1.14 (1.09–1.19)
Tertiles of CV (%)					
T1 (0–6.47)	21/621	3.4	1(ref.)	1(ref.)	1(ref.)
T2 (6.47–10.29)	32/616	5.2	1.49 (0.85–2.63)	1.14 (0.62–2.11)	1.07 (0.58–1.98)
T3 (10.29–47.20)	100/619	16.2	5.39 (3.31–8.77)	3.11 (1.82–5.30)	2.38 (1.36–4.17)
*P* for trend		<.001	<.001	<.001	<.001
Each 1 increment			1.20 (1.16–1.23)	1.15 (1.11–1.20)	1.13 (1.09–1.18)
Tertiles of ASV (mg/dl)					
T1 (0–6.58)	17/635	2.7	1(ref.)	1(ref.)	1(ref.)
T2 (6.58–10.63)	25/594	4.2	1.57 (0.84–2.94)	1.17 (0.60–2.28)	1.12 (0.58–2.19)
T3 (10.63–66.76)	111/627	17.7	7.59 (4.48–12.85)	3.41 (1.92–6.06)	2.70 (1.49–4.88)
*P* for trend		<.001	<.001	<.001	<.001
Each 1 increment			1.14 (1.11–1.16)	1.10 (1.07–1.13)	1.08 (1.05–1.12)
Tertiles of VIM (%)					
T1 (0–1.26)	19/622	3.1	1(ref.)	1(ref.)	1(ref.)
T2 (1.26–2.02)	28/615	4.6	1.43 (0.79–2.61)	1.18 (0.62–2.23)	1.13 (0.60–2.14)
T3 (2.02–13.08)	106/619	17.1	6.33 (3.82–10.49)	2.99 (1.72–5.20)	2.29 (1.29–4.08)
*P* for trend		<.001	<.001	<.001	.002
Each 1 increment			2.67 (2.28–3.13)	2.00 (1.66–2.41)	1.84 (1.51–2.26)

*Note*: Model 1: adjusted for age, sex, waist circumferences, diabetes family history, alcohol drinking, smoking, regular exercise.

*Note*: Model 2: adjusted for model 1 plus baseline SBP, LDL‐c, log_10_TG, prediabetes, antihypertensive medications, lipid‐lowering medications, and mean FPG at three visits.

*Note*: Model 3: adjusted for model 2 plus ΔHOMA‐IR.

Abbreviations: ASV, the average successive variability; CI, confidence interval; CV, coefficient of variation; FPG, fasting plasma glucose; HOMA‐IR, homeostatic model assessment of insulin resistance index; LDL‐c, low‐density lipoprotein cholesterol; log_10_TG, log_10_ transformed triglycerides; OR, odds ratio; SBP, systolic blood pressure; VVV, visit‐to‐visit variability; VIM, the variability independent of the mean.

We further explored the effects of VVV in FPG on incident diabetes by increasing or decreasing HOMA‐IR between the second and the third visits (Supplementary Tables S4–S7 in Appendix S1). We found that the multivariable‐adjusted OR for the highest tertile of FPG‐SD was significantly associated with incident diabetes in individuals with increased HOMA‐IR (OR = 5.13; 95% CI, 1.72–15.33) but not in those with decreased HOMA‐IR. The highest tertile of FPG‐CV, FPG‐ASV, and FPG‐VIM were also significantly associated with diabetes risks only in individuals with increasing HOMA‐IR.

### 3.3 Association of ΔHOMA‐IR with VVV in FPG

The relations of VVV in FPG with ΔHOMA‐IR based on generalized linear regression models are presented in Table 3. After multivariable adjustment including mean FPG at three3 visits, participants in the highest tertile of all four FPG variability indices showed significantly elevated ΔHOMA‐IR (all *p* < .001), compared with those in the lowest tertile (0–5.83 mg/dl). The highest tertile of FPG‐SD (9.55–74.17 mg/dl) showed significantly elevated ΔHOMA‐IR (β = 0.55; 95% CI, 0.45–0.65) compared with the lowest tertile. Similar results were observed with other measures of VVV in FPG including FPG‐CV (β = 0.54; 95% CI, 0.45–0.64), FPG‐ASV (β = 0.51; 95% CI, 0.41–0.61), and FPG‐VIM (β = 0.56; 95% CI, 0.46–0.66).

**TABLE 3:** Multivariable‐adjusted associations of tertiles of VVV in FPG with ΔHOMA‐IR

	β (95% CI)					
	Model 1	*P* value	Model 2	*P* value	Model 3	*P* value
Tertiles of SD (mg/dl)						
T1 (0–5.83)	1(ref.)		1(ref.)		1(ref.)	
T2 (5.83–9.55)	0.10 (0.01–0.20)	.038	0.10 (0.00–0.19)	.049	0.10 (0.01–0.20)	.034
T3 (9.55–74.17)	0.51 (0.41–0.60)	<.001	0.52 (0.42–0.62)	<.001	0.55 (0.45–0.65)	<.001
Tertiles of CV (%)						
T1 (0–6.47)	1(ref.)		1(ref.)		1(ref.)	
T2 (6.47–10.29)	0.12 (0.03–0.22)	.011	0.12 (0.03–0.22)	.012	0.12 (0.03–0.22)	.011
T3 (10.29–47.20)	0.53 (0.44–0.63)	<.001	0.54 (0.44–0.63)	<.001	0.54 (0.45–0.64)	<.001
Tertiles of ASV (mg/dl)						
T1 (0–6.58)	1(ref.)		1(ref.)		1(ref.)	
T2 (6.58–10.63)	0.16 (0.06–0.25)	.001	0.16 (0.06–0.25)	.001	0.16 (0.07–0.26)	.001
T3 (10.63–66.76)	0.48 (0.39–0.58)	<.001	0.50 (0.40–0.59)	<.001	0.51 (0.41–0.61)	<.001
Tertiles of VIM (%)						
T1 (0–1.26)	1(ref.)		1(ref.)		1(ref.)	
T2 (1.26–2.02)	0.10 (0.01–0.20)	.036	0.10 (0.00–0.19)	.044	0.10 (0.01–0.20)	.034
T3 (2.02–13.08)	0.53 (0.43–0.62)	<.001	0.54 (0.44–0.63)	<.001	0.56 (0.46–0.66)	<.001

*Note*: Model 1: adjusted for age, sex, waist circumferences, diabetes family history, alcohol drinking, smoking, regular exercise.

*Note*: Model 2: adjusted for model 1 plus baseline SBP, LDL‐c, log_10_TG, prediabetes, antihypertensive medications, and lipid‐lowering medications.

*Note*: Model 3: adjusted for model 2 plus mean FPG at three visits.

Abbreviations: ASV, the average successive variability; CI, confidence interval; CV, coefficient of variation; FPG, fasting plasma glucose; HOMA‐IR, homeostatic model assessment of insulin resistance index; LDL‐c, low‐density lipoprotein cholesterol; log_10_TG, log_10_ transformed triglycerides; SBP, systolic blood pressure; VIM, the variability independent of the mean; VVV, visit‐to‐visit variability; β, regression coefficient.

### 3.4 Association of ΔHOMA‐IR with incident diabetes

The multivariable logistic regression analysis showed that ΔHOMA‐IR was significantly and independently associated with incident diabetes even after full adjustment for confounders including mean FPG at three visits and HOMA‐IR at the second visit (OR = 1.81; 95% CI, 1.46–2.25; Supplementary Table S8 in Appendix S1).

### 3.5 Mediation analyses

**FIGURE 2:**
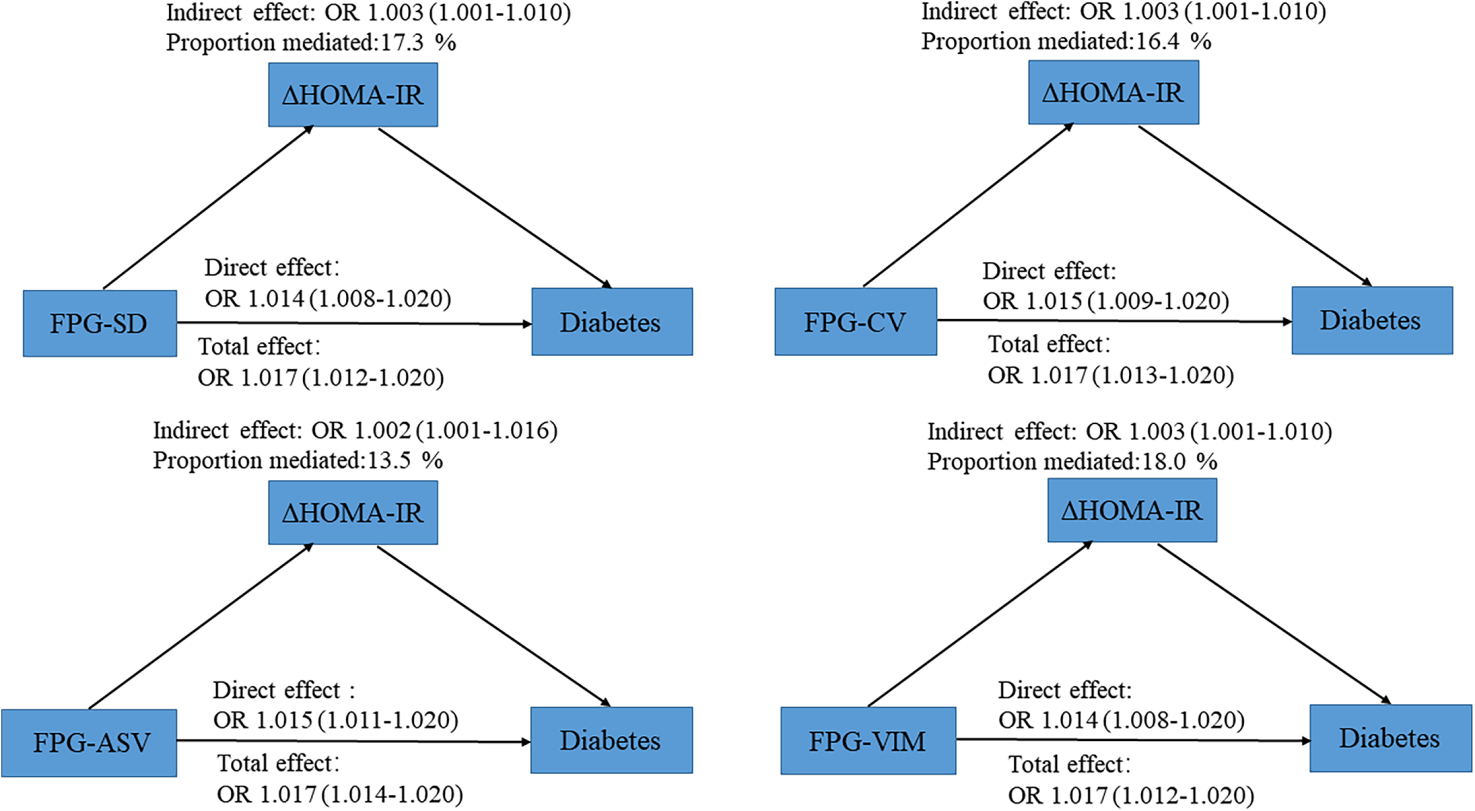
Mediation analysis of ΔHOMA‐IR in the association between VVV in FPG and incident diabetes. Adjusted for age, sex, waist circumferences, diabetes family history, alcohol drinking, smoking, regular exercise, baseline SBP, LDL‐c, log_10_TG, prediabetes, antihypertensive medications, lipid‐lowering medications, and mean FPG at three visits. ASV, the average successive variability; CI, confidence interval; CV, coefficient of variation; FPG, fasting plasma glucose; HOMA‐IR, homeostatic model assessment of insulin resistance index; LDL‐c, low‐density lipoprotein cholesterol; log_10_TG, log_10_ transformed triglycerides; OR, odds ratios; SBP, systolic blood pressure; VIM, the variability independent of the mean; VVV, visit‐to‐visit variability

Findings from the mediation analyses are presented in Figure 2. After adjustment for confounding factors including mean FPG at three visits, a significant mediation was observed for the association between FPG‐SD and diabetes risk by ΔHOMA‐IR (indirect effect: OR = 1.003; 95% CI, 1.001–1.010; proportion mediated: 17.3%). Similar results were observed with other measures of VVV in FPG including FPG‐CV (indirect effect: OR = 1.003; 95% CI, 1.001–1.010; proportion mediated: 16.4%), FPG‐ASV (indirect effect: OR = 1.002; 95% CI, 1.001–1.016; proportion mediated: 13.5%), and FPG‐VIM (indirect effect: OR = 1.003; 95% CI, 1.001–1.010; proportion mediated: 18.0%).

## 4 DISCUSSION

In the current study, we found that the high variabilities in FPG assessed by four indicators (SD, CV, ASV, and VIM) were associated with increased risks of diabetes in a Chinese population of community residents aged ≥40 years. The association remained significant after multivariable adjustment for traditional risk factors including mean level of FPG at three visits. In addition, we observed a significant mediation of the association by changes in HOMA‐IR, indicating that changes in insulin sensitivity might be a potential mechanism underlying the association between glucose variabilities and diabetes risks.

Recently, glycemic variability has drawn attention because it was reported that glycemic variability might be a better predictor of complications than average glycemic levels in people with diabetes.^22^ The Verona Diabetes Study revealed that mean FPG level was not a predictor of total mortality after adjustment for long‐term variability of FPG.^23^ The post hoc analysis of the ADVANCE (Action in Diabetes and Vascular Disease: Preterax and Diamicron MR Controlled Evaluation) trial demonstrated that VVV in HbA1c as well as VVV in fasting glucose were significantly associated with macrovascular events, microvascular events, and all‐cause mortality independent of cardiovascular risk factors, including cumulative mean HbA1c or fasting glucose.^5^ Furthermore, a recent meta‐analysis suggested that greater HbA1c variability is associated with several microvascular and macrovascular complications and mortality in patients with diabetes independent of the HbA1c level.^24^

Unlike these prior studies, our study examined the association between the long‐term glycemic variability and development of diabetes in a general Chinese population. Our findings suggested that the VVV in FPG may play an important role in the development of diabetes. It was in line with a recent study using data from the Coronary Artery Risk Development in Young Adults (CARDIA) showing that higher FPG variability during young adulthood without diabetes was significantly associated with incident diabetes.^9^ As was found in the current study, compared with the lowest tertile of FPG‐SD, the highest tertile was associated with a 148% increased risk for diabetes, after adjustment for confounding factors including mean FPG at three visits.

There are several potential pathophysiological mechanisms that link glucose variability with incident diabetes. Previous studies in animals and humans have shown that intermittent high blood glucose exposure is more detrimental than stable hyperglycemia in activation of inflammation, generation of oxidative stress, vascular endothelial dysfunction, and incident IR, all of which may promote the development of diabetes.^25^, ^26^, ^27^, ^28^ The significant medication of the association between FPG variability and diabetes risk by changes in HOMA‐IR, as found in the current study, provided further evidence that glucose variability leads to increased diabetes risk through worsening of insulin sensitivity. To the best of our knowledge, this study was the first to explore mediating role of ΔHOMA‐IR in the relation between FPG variability and incident diabetes.

Findings from the current study have important public health and clinical implications. With the development of new technologies, glucose variability as an additional index for glycemic control will be used more commonly by physicians.^22^ It is noteworthy that the International Consensus on Use of Continuous Glucose Monitoring has recently integrated a glucose variability (CV) target of <36% as a key metric for defining stable glucose levels in diabetes.^22^, ^29^ In addition, studies have suggested that a lower glycemic variability (CV) target of <33% provides additional protection against hypoglycemia for those receiving insulin or sulfonylureas.^30^, ^31^ Therefore, in addition to a regular monitoring of FPG levels, an evaluation of the FPG variabilities should also be considered in early prevention of diabetes among the general population.

Our study has several strengths. A comprehensive evaluation of FPG variability was assessed by four indicators (SD, CV, ASV, and VIM). An extensive adjustment for traditional risk factors was done, including adjustment for mean levels of FPG at three visits. In addition, the potential role of ΔHOMA‐IR as a mediator was first evaluated in a general population, providing population data for the underlying mechanism of glycemic variability leading to diabetes. Nevertheless, there are also limitations. First, OGTT was not conducted at the first visit, which limited detailed and further subtyping of glucose status. Second, ΔHOMA‐IR was assessed between the second and the third visits, which could smooth possible changes in HOMA‐IR and thus possibly limit its mediating effect. Third, the long‐term glycemic variability was calculated using FPG levels at all three visits and a prospective analysis with incident diabetes after the third visit should have been used to better elucidate the potential causal relationship between glycemic variability and diabetes. Fourth, our study was conducted in middle‐aged and elderly Chinese adults aged ≥40 years; therefore, the results may not be generalizable to other ethnic or age groups.

## 5 CONCLUSION

Our findings suggest that a greater variability in FPG is significantly associated with an increased risk of incident diabetes, independent of mean levels of FPG among middle‐aged and older Chinese adults. The association could be partially mediated by an elevated HOMA‐IR. Future prospective studies are needed to demonstrate whether medications or treatment in reducing glycemic variabilities is a potential therapeutic target for diabetes prevention and management.

## DISCLOSURE

The authors have no conflicts of interest to disclose.

## Supporting information


**Appendix** **S1**. Supplementary Information
