# Supplementary material for: Impact of visit‐to‐visit fasting plasma glucose variability on the development of diabetes: The mediation by insulin resistance
Source: J Diabetes. 2022 Feb 16;14(3):205–15. doi: 10.1111/1753-0407.13253 (PMC9060060; doi:10.1111/1753-0407.13253)
Supplement: Supplementary file 1 — Appendix S1. Supplementary Information [file JDB-14-205-s001.zip › JDB_13253_Supplementary-JDB-2021-506-R1.docx]

Supplementary Material

Impact of visit-to-visit fasting plasma glucose variability on the development of diabetes: the mediation by insulin resistance

Supplementary Figure 1. Changes of fasting plasma glucose levels at the three visits.


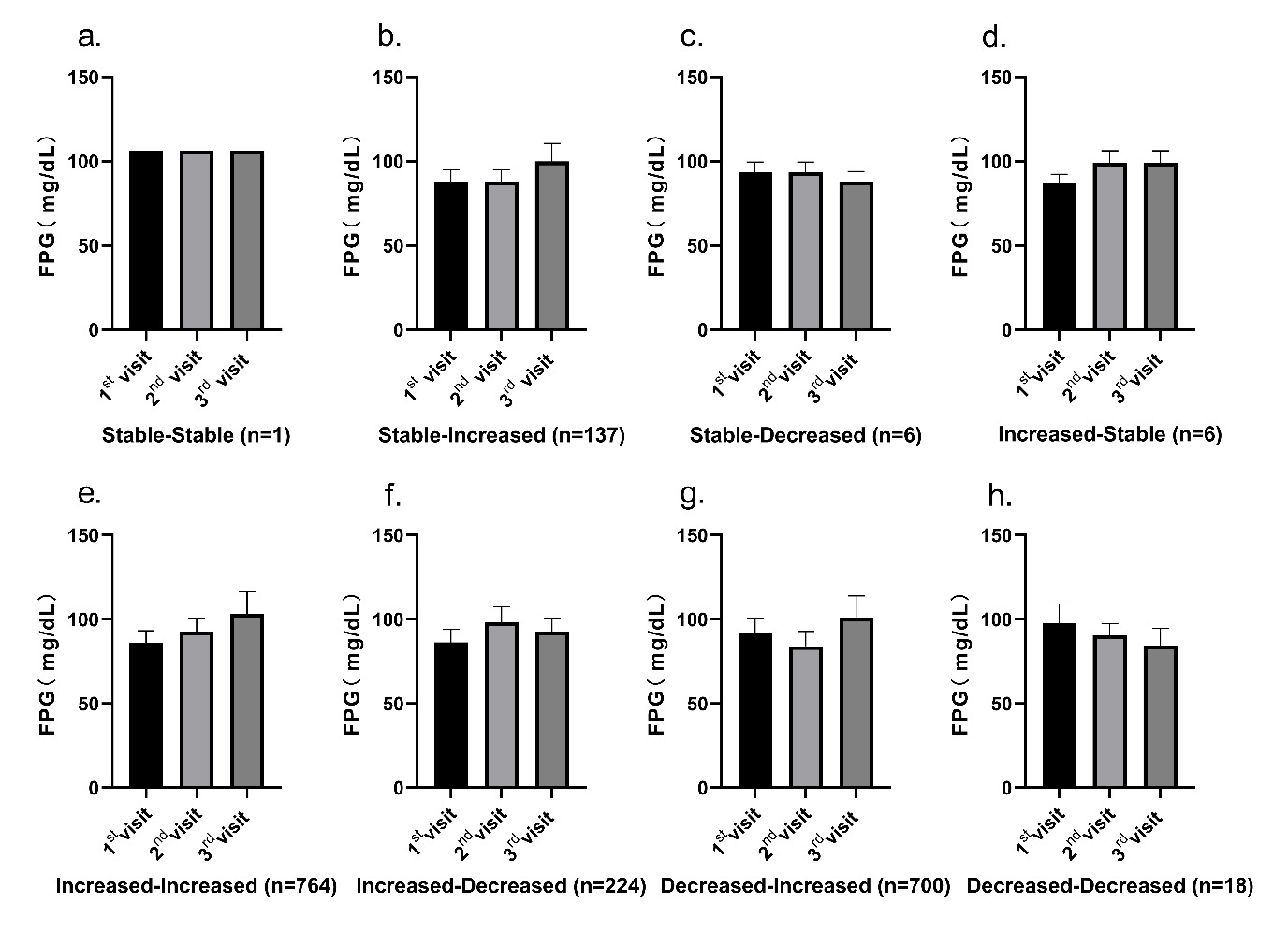


Supplementary Table 1. Characteristics of participants according to the tertiles of the FPG variability (FPG-CV)

|  | T1 (0-6.47 %) | T2 (6.47-10.29 %) | T3 (10.29-47.20 %) | *P* value |
| --- | --- | --- | --- | --- |
| Participants, n | 621 | 616 | 619 |  |
| Age (years) | 57.5 ± 8.9 | 58.1 ± 9.5 | 57.8 ± 8.9 | 0.468 |
| Men, n (%) | 209 (33.7) | 217 (35.2) | 240 (38.8) | 0.157 |
| Family history of diabetes, n (%) | 84 (13.6) | 86 (14.0) | 88 (14.2) | 0.943 |
| BMI (kg/m^2^) | 24.6 ± 3.3 | 24.8 ± 3.6 | 25.1 ± 3.7 | 0.036 |
| Waist circumference (cm) | 82.8 ± 9.1 | 83.4 ± 9.7 | 83.9 ± 9.4 | 0.118 |
| Lifestyle factors, n (%) |  |  |  |  |
| Current smoking | 146 (23.5) | 142 (23.1) | 150 (24.2) | 0.886 |
| Current drinking | 100 (16.1) | 101 (16.4) | 116 (18.7) | 0.401 |
| Regular exercise | 34 (5.5) | 28 (4.6) | 36 (5.8) | 0.587 |
| Blood pressure (mmHg) |  |  |  |  |
| SBP | 129 ± 21 | 129 ± 20 | 133 ± 20 | < 0.001 |
| DBP | 79 ± 10 | 78 ± 10 | 80 ± 10 | < 0.001 |
| Lipid profile (mg/dL) |  |  |  |  |
| TG | 99.3 (70.7-136.8) | 101.5 (75.2-149.3) | 102.3 (70.7-148.9) | 0.227 |
| TC | 196.3 ± 32.9 | 197.5 ± 33.9 | 196.5 ± 36.4 | 0.813 |
| HDL-c | 55.1 ± 11.0 | 54.3 ± 11.5 | 54.5 ± 12.2 | 0.510 |
| LDL-c | 94.1 ± 25.3 | 95.4 ± 24.2 | 93.5 ± 24.9 | 0.371 |
| Fasting serum insulin, at the 2^nd^ visit (μIU/mL) | 6.4 (4.3-8.9) | 6.5 (4.2-9.4) | 6.7 (4.4-10.4) | 0.082 |
| Fasting serum insulin, at the 3^rd^ visit (μIU/mL) | 5.9 (4.4-7.8) | 6.2 (4.4-8.2) | 7.3 (5.2-10.0) | < 0.001 |
| HOMA-IR at the 2^nd^ visit | 1.4 (0.9-2.0) | 1.4 (0.9-2.2) | 1.5 (0.91-2.3) | 0.380 |
| HOMA-IR at the 3^rd^ visit | 1.4 (1.0-1.9) | 1.5 (1.1-2.0) | 1.9 (1.3-2.7) | < 0.001 |
| Δ HOMA-IR | -0.04 (-0.42-0.29) | 0.10 (-0.45-0.45) | 0.41 (-0.09-0.93) | < 0.001 |
| FPG at baseline (mg/dL) | 89.2 ± 7.2 | 87.8 ± 8.3 | 88.1 ± 9.8 | 0.009 |
| FPG variability |  |  |  |  |
| SD (mg/dL) | 4.0 ± 1.4 | 7.7 ± 1.3 | 14.0 ± 5.8 | < 0.001 |
| CV (%) | 4.3 ± 1.5 | 8.3 ± 1.1 | 14.6 ± 4.6 | < 0.001 |
| ASV (mg/dL) | 4.7 ± 2.0 | 8.7 ± 2.4 | 15.8 ± 6.9 | < 0.001 |
| VIM (%) | 0.84 ± 0.29 | 1.6 ± 0.24 | 2.9 ± 1.1 | < 0.001 |
| Hypertension, n (%) | 225 (36.2) | 219 (35.6) | 276 (44.7) | 0.001 |
| Dyslipidemia, n (%) | 140 (22.5) | 153 (24.8) | 175 (28.3) | 0.065 |
| Medications |  |  |  |  |
| Antihypertensive medications at any of the 3 visits, n (%) | 151 (24.3) | 143 (23.2) | 206 (33.3) | < 0.001 |
| Lipid-lowering medications at any of the 3 visits, n (%) | 3 (0.48) | 6 (0.97) | 7 (1.1) | 0.437 |

Data are means ± SDs or medians (IQRs) for continuous variables, or numbers (percentages) for categorical variables.

BMI: body mass index; SBP: systolic blood pressure; DBP: diastolic blood pressure; TC: total cholesterol; TG: triglyceride; HDL-c: high-density lipoprotein cholesterol; LDL-c: low-density lipoprotein cholesterol; HOMA-IR: homeostatic model assessment of insulin resistance index; FPG: fasting plasma glucose; SD: standard deviation; CV, coefficient of variation; ASV: the average successive variability; VIM: variability independent of the mean; IQR, interquartile range.

There were 1 missing value for family history of diabetes, 1 missing value for BMI, 2 missing values for SBP, 2 missing values for DBP, and 1 missing value for hypertension, respectively.

Supplementary Table 2. Characteristics of participants according to the tertiles of the FPG variability (FPG-ASV)

|  | T1 (0-6.58 mg/dL) | T2 (6.58-10.63 mg/dL) | T3 (10.63-66.76 mg/dL) | *P* value |
| --- | --- | --- | --- | --- |
| Participants, n | 635 | 594 | 627 |  |
| Age (years) | 57.3 ± 8.8 | 57.8 ± 9.5 | 58.2 ± 9.0 | 0.205 |
| Men, n (%) | 225 (35.4) | 187 (31.5) | 254 (40.5) | 0.004 |
| Family history of diabetes, n (%) | 89 (14.0) | 84 (14.1) | 85 (13.6) | 0.951 |
| BMI (kg/m^2^) | 24.6 ± 3.3 | 24.7 ± 3.5 | 25.2 ± 3.7 | 0.008 |
| Waist circumference (cm) | 82.8 ± 9.2 | 83.0 ± 9.6 | 84.3 ± 9.4 | 0.010 |
| Lifestyle factors, n (%) |  |  |  |  |
| Current smoking | 160 (25.2) | 122 (20.5) | 156 (24.9) | 0.103 |
| Current drinking | 107 (16.9) | 92 (15.5) | 118 (18.8) | 0.297 |
| Regular exercise | 36 (5.7) | 27 (4.6) | 35 (5.6) | 0.623 |
| Blood pressure (mmHg) |  |  |  |  |
| SBP | 128 ± 20 | 129 ± 20 | 133 ± 21 | < 0.001 |
| DBP | 78 ± 10 | 78 ± 10 | 80 ± 10 | < 0.001 |
| Lipid profile (mg/dL) |  |  |  |  |
| TG | 100.8 (70.7-141.4) | 99.3 (72.9-145.1) | 103.0 (74.4-151.1) | 0.351 |
| TC | 196.6 ± 32.6 | 197.5 ± 34.1 | 196.1 ± 36.5 | 0.765 |
| HDL-c | 54.9 ± 11.2 | 54.9 ± 11.5 | 54.2 ± 12.0 | 0.413 |
| LDL-c | 94.4 ± 24.8 | 95.2 ± 24.9 | 93.4 ± 24.7 | 0.450 |
| Fasting serum insulin, at the 2^nd^ visit (μIU/mL) | 6.4 (4.2-8.9) | 6.2 (4.4-9.3) | 6.8 (4.4-10.4) | 0.037 |
| Fasting serum insulin, at the 3^rd^ visit (μIU/mL) | 5.8 (4.3-7.8) | 6.4 (4.7-8.4) | 7.1 (5.0-10.0) | < 0.001 |
| HOMA-IR at the 2^nd^ visit | 1.4 (0.9-2.0) | 1.4 (1.0-2.2) | 1.5 (0.90-2.4) | 0.120 |
| HOMA-IR at the 3^rd^ visit | 1.3 (1.0-1.9) | 1.6 (1.1-2.1) | 1.9 (1.3-2.7) | < 0.001 |
| Δ HOMA-IR | -0.03 (-0.39-0.30) | 0.12 (-0.37-0.52) | 0.38 (-0.14-0.91) | < 0.001 |
| FPG at baseline (mg/dL) | 87.7 ± 7.2 | 87.0 ± 7.4 | 90.4 ± 10.2 | < 0.001 |
| FPG variability |  |  |  |  |
| SD (mg/dL) | 4.2 ± 1.6 | 7.9 ± 1.8 | 13.6 ± 6.0 | < 0.001 |
| CV (%) | 4.7 ± 1.8 | 8.5 ± 1.9 | 14.0 ± 5.0 | < 0.001 |
| ASV (mg/dL) | 4.4 ± 1.5 | 8.4 ± 1.1 | 16.3 ± 6.5 | < 0.001 |
| VIM (%) | 0.90 ± 0.35 | 1.7 ± 0.37 | 2.8 ± 1.2 | < 0.001 |
| Hypertension, n (%) | 221 (34.8) | 213 (35.9) | 286 (45.7) | < 0.001 |
| Dyslipidemia, n (%) | 148 (23.3) | 140 (23.6) | 180 (28.7) | 0.047 |
| Medications |  |  |  |  |
| Antihypertensive medications at any of the 3 visits, n (%) | 137 (21.6) | 158 (26.6) | 205 (32.7) | < 0.001 |
| Lipid-lowering medications at any of the 3 visits, n (%) | 3 (0.47) | 6 (1.0) | 7 (1.1) | 0.416 |

Data are means ± SDs or medians (IQRs) for continuous variables, or numbers (percentages) for categorical variables.

BMI: body mass index; SBP: systolic blood pressure; DBP: diastolic blood pressure; TC: total cholesterol; TG: triglyceride; HDL-c: high-density lipoprotein cholesterol; LDL-c: low-density lipoprotein cholesterol; HOMA-IR: homeostatic model assessment of insulin resistance index; FPG: fasting plasma glucose; SD: standard deviation; CV, coefficient of variation; ASV: the average successive variability; VIM: variability independent of the mean; IQR, interquartile range.

There were 1 missing value for family history of diabetes, 1 missing value for BMI, 2 missing values for SBP, 2 missing values for DBP, and 1 missing value for hypertension, respectively.

Supplementary Table 3. Characteristics of participants according to the tertiles of the FPG variability (FPG-VIM)

|  | T1 (0-1.26 %) | T2 (1.26-2.02 %) | T3 (2.02-13.08 %) | *P* value |
| --- | --- | --- | --- | --- |
| Participants, n | 622 | 615 | 619 |  |
| Age (years) | 57.5 ± 8.8 | 57.8 ± 9.6 | 58.0 ± 8.9 | 0.531 |
| Men, n (%) | 214 (34.4) | 210 (34.2) | 242 (39.1) | 0.124 |
| Family history of diabetes, n (%) | 83 (13.4) | 87 (14.2) | 88 (14.2) | 0.891 |
| BMI (kg/m^2^) | 24.6 ± 3.3 | 24.8 ± 3.6 | 25.1 ± 3.6 | 0.023 |
| Waist circumference (cm) | 82.6 ± 9.0 | 83.5 ± 9.8 | 84.0 ± 9.4 | 0.035 |
| Lifestyle factors, n (%) |  |  |  |  |
| Current smoking | 151 (24.3) | 139 (22.6) | 148 (23.9) | 0.767 |
| Current drinking | 100 (16.1) | 103 (16.8) | 114 (18.4) | 0.530 |
| Regular exercise | 34 (5.5) | 29 (4.7) | 35 (5.7) | 0.738 |
| Blood pressure (mmHg) |  |  |  |  |
| SBP | 129 ± 21 | 129 ± 20 | 133 ± 20 | < 0.001 |
| DBP | 79 ± 10 | 78 ± 10 | 81 ± 10 | < 0.001 |
| Lipid profile (mg/dL) |  |  |  |  |
| TG | 100.4 (70.7-139.9) | 99.3 (74.4-147.4) | 103.8 (71.4-151.1) | 0.342 |
| TC | 196.0 ± 32.9 | 197.5 ± 33.9 | 196.7 ± 36.5 | 0.755 |
| HDL-c | 55.0 ± 10.9 | 54.6 ± 11.5 | 54.3 ± 12.2 | 0.572 |
| LDL-c | 94.0 ± 25.1 | 95.3 ± 24.4 | 93.6 ± 24.9 | 0.452 |
| Fasting serum insulin, at the 2^nd^ visit (μIU/mL) | 6.4 (4.3-8.9) | 6.5 (4.2-9.4) | 6.8 (4.5-10.4) | 0.027 |
| Fasting serum insulin, at the 3^rd^ visit (μIU/mL) | 5.8 (4.4-7.8) | 6.1 (4.3-8.2) | 7.3 (5.3-10.0) | < 0.001 |
| HOMA-IR at the 2^nd^ visit | 1.4 (0.9-2.0) | 1.4 (0.9-2.2) | 1.5 (0.93-2.3) | 0.047 |
| HOMA-IR at the 3^rd^ visit | 1.3 (1.0-1.9) | 1.5 (1.1-2.0) | 2.0 (1.4-2.7) | < 0.001 |
| Δ HOMA-IR | -0.03 (-0.39-0.29) | 0.10 (-0.45-0.45) | 0.42 (-0.10-0.94) | < 0.001 |
| FPG at baseline (mg/dL) | 88.7 ± 7.3 | 87.5 ± 8.0 | 89.0 ± 10.0 | 0.008 |
| FPG variability |  |  |  |  |
| SD (mg/dL) | 3.9 ± 1.4 | 7.7 ± 1.1 | 14.0 ± 5.7 | < 0.001 |
| CV (%) | 4.4 ± 1.5 | 8.4 ± 1.2 | 14.5 ± 4.7 | < 0.001 |
| ASV (mg/dL) | 4.7 ± 2.0 | 8.7 ± 2.4 | 15.8 ± 6.9 | < 0.001 |
| VIM (%) | 0.84 ± 0.29 | 1.6 ± 0.22 | 2.9 ± 1.1 | < 0.001 |
| Hypertension, n (%) | 224 (36.0) | 213 (34.6) | 283 (45.8) | < 0.001 |
| Dyslipidemia, n (%) | 140 (22.5) | 152 (24.7) | 176 (28.4) | 0.052 |
| Medications |  |  |  |  |
| Antihypertensive medications at any of the 3 visits, n (%) | 153 (24.6) | 138 (22.4) | 209 (33.8) | < 0.001 |
| Lipid-lowering medications at any of the 3 visits, n (%) | 3 (0.48) | 6 (0.98) | 7 (1.1) | 0.435 |

Data are means ± SDs or medians (IQRs) for continuous variables, or numbers (percentages) for categorical variables.

BMI: body mass index; SBP: systolic blood pressure; DBP: diastolic blood pressure; TC: total cholesterol; TG: triglyceride; HDL-c: high-density lipoprotein cholesterol; LDL-c: low-density lipoprotein cholesterol; HOMA-IR: homeostatic model assessment of insulin resistance index; FPG: fasting plasma glucose; SD: standard deviation; CV, coefficient of variation; ASV: the average successive variability; VIM: variability independent of the mean; IQR, interquartile range.

There were 1 missing value for family history of diabetes, 1 missing value for BMI, 2 missing values for SBP, 2 missing values for DBP, and 1 missing value for hypertension, respectively.

Supplementary Table 4. Association of VVV in FPG using SD with incident diabetes by increased or decreased HOMA-IR between the 2^nd^ and the 3^rd^ visits

|  | Incident cases/  No. of participants | Cumulative  incidence (%) | OR (95% CI) | | |
| --- | --- | --- | --- | --- | --- |
|  |  |  | Model 1 | Model 2 | Model 3 |
| Increased HOMA-IR |  |  |  |  |  |
| Tertiles of SD (mg/dL) |  |  |  |  |  |
| T1 (0-5.83) | 4/296 | 1.4 | 1(ref.) | 1(ref.) | 1(ref.) |
| T2 (5.83-9.55) | 11/344 | 3.2 | 2.26 (0.71-7.20) | 2.32 (0.68-7.88) | 2.13 (0.63-7.18) |
| T3 (9.55-74.17) | 87/439 | 19.8 | 17.13 (6.19-47.40) | 6.64 (2.25-19.60) | 5.13 (1.72-15.33) |
| *P* for trend | / | < 0.001 | < 0.001 | < 0.001 | < 0.001 |
| Each 1 increment | / | / | 1.29 (1.23-1.35) | 1.20 (1.13-1.27) | 1.18 (1.11-1.25) |
| Decreased HOMA-IR | | | | | |
| Tertiles of SD (mg/dL) |  |  |  |  |  |
| T1 (0-5.83) | 13/324 | 4.0 | 1(ref.) | 1(ref.) | 1(ref.) |
| T2 (5.83-9.55) | 15/274 | 5.5 | 1.28 (0.59-2.77) | 1.09 (0.47-2.52) | 1.09 (0.47-2.51) |
| T3 (9.55-74.17) | 23/179 | 12.9 | 3.27 (1.60-6.68) | 1.42 (0.62-3.27) | 1.42 (0.62-3.27) |
| *P* for trend | / | < 0.001 | 0.001 | 0.408 | 0.408 |
| Each 1 increment | / | / | 1.15 (1.08-1.21) | 1.08 (1.01-1.16) | 1.08 (1.01-1.16) |

Model 1: adjusted for age, sex, waist circumferences, diabetes family history, alcohol drinking, smoking, regular exercise.

Model 2: adjusted for model 1 plus baseline SBP, LDL-c, log_10_TG, prediabetes, antihypertensive medications, lipid-lowering medications, and mean FPG at 3 visits.

Model 3: adjusted for model 2 plus ΔHOMA-IR.

VVV, visit-to-visit variability; OR, odds ratios; CI, confidence interval; FPG, fasting plasma glucose; SD, standard deviation; SBP, systolic blood pressure; LDL-c, low-density lipoprotein cholesterol; log_10_TG, log_10_ transformed triglycerides; HOMA-IR, homeostatic model assessment of insulin resistance index.

Supplementary Table 5. Association of VVV in FPG using CV with incident diabetes by increased or decreased HOMA-IR between the 2^nd^ and the 3^rd^ visits

| ΔHOMA-IR | Incident cases/  No. of participants | Cumulative  incidence (%) | OR (95% CI) | | |
| --- | --- | --- | --- | --- | --- |
|  |  |  | Model 1 | Model 2 | Model 3 |
| Increased HOMA-IR |  |  |  |  |  |
| Tertiles of CV (%) |  |  |  |  |  |
| T1 (0-6.47) | 5/289 | 1.7 | 1(ref.) | 1(ref.) | 1(ref.) |
| T2 (6.47-10.29) | 16/343 | 4.7 | 2.58 (0.93-7.17) | 2.44 (0.82-7.26) | 2.22 (0.75-6.59) |
| T3 (10.29-47.20) | 81/447 | 18.1 | 11.92 (4.75-29.92) | 6.36 (2.36-17.09) | 4.89 (1.79-13.34) |
| *P* for trend | / | < 0.001 | < 0.001 | < 0.001 | < 0.001 |
| Each 1 increment | / | / | 1.24 (1.19-1.29) | 1.20 (1.13-1.26) | 1.18 (1.11-1.24) |
| Decreased HOMA-IR |  |  |  |  |  |
| Tertiles of CV (%) |  |  |  |  |  |
| T1 (0-6.47) | 16/332 | 4.8 | 1(ref.) | 1(ref.) | 1(ref.) |
| T2 (6.47-10.29) | 16/273 | 5.9 | 1.14 (0.55-2.34) | 0.80 (0.36-1.79) | 0.80 (0.36-1.79) |
| T3 (10.29-47.20) | 19/172 | 11.1 | 2.30 (1.14-4.65) | 1.42 (0.64-3.16) | 1.41 (0.63-3.15) |
| *P* for trend | / | 0.024 | 0.024 | 0.433 | 0.434 |
| Each 1 increment | / | / | 1.12 (1.07-1.18) | 1.07 (1.00-1.15) | 1.07 (1.00-1.15) |

Model 1: adjusted for age, sex, waist circumferences, diabetes family history, alcohol drinking, smoking, regular exercise.

Model 2: adjusted for model 1 plus baseline SBP, LDL-c, log_10_TG, prediabetes, antihypertensive medications, lipid-lowering medications, and mean FPG at 3 visits.

Model 3: adjusted for model 2 plus ΔHOMA-IR.

VVV, visit-to-visit variability; OR, odds ratios; CI, confidence interval; FPG, fasting plasma glucose; CV, coefficient of variation; SBP, systolic blood pressure; LDL-c, low-density lipoprotein cholesterol; log_10_TG, log_10_ transformed triglycerides; HOMA-IR, homeostatic model assessment of insulin resistance index.

Supplementary Table 6. Association of VVV in FPG using ASV with incident diabetes by increased or decreased HOMA-IR between the 2^nd^ and the 3^rd^ visits

| ΔHOMA-IR | Incident cases/  No. of participants | Cumulative  incidence (%) | OR (95% CI) | | |
| --- | --- | --- | --- | --- | --- |
|  |  |  | Model 1 | Model 2 | Model 3 |
| Increased HOMA-IR |  |  |  |  |  |
| Tertiles of ASV (mg/dL) |  |  |  |  |  |
| T1 (0-6.58) | 5/304 | 1.6 | 1(ref.) | 1(ref.) | 1(ref.) |
| T2 (6.58-10.63) | 12/339 | 3.5 | 2.18 (0.76-6.30) | 2.26 (0.74-6.91) | 2.02 (0.66-6.16) |
| T3 (10.63-66.76) | 85/436 | 19.5 | 13.91 (5.54-34.89) | 5.88 (2.20-15.73) | 4.47 (1.64-12.21) |
| *P* for trend | / | < 0.001 | < 0.001 | < 0.001 | 0.001 |
| Each 1 increment | / | / | 1.16 (1.12-1.19) | 1.12 (1.08-1.17) | 1.11 (1.06-1.15) |
| Decreased HOMA-IR |  |  |  |  |  |
| Tertiles of ASV (mg/dL) |  |  |  |  |  |
| T1 (0-6.58) | 12/331 | 3.6 | 1(ref.) | 1(ref.) | 1(ref.) |
| T2 (6.58-10.63) | 13/255 | 5.1 | 1.32 (0.59-2.97) | 0.84 (0.34-2.04) | 0.84 (0.34-2.04) |
| T3 (10.63-66.76) | 26/191 | 13.6 | 4.02 (1.96-8.25) | 1.84 (0.81-4.17) | 1.84 (0.81-4.18) |
| *P* for trend | / | < 0.001 | < 0.001 | 0.111 | 0.113 |
| Each 1 increment | / | / | 1.10 (1.05-1.14) | 1.04 (0.98-1.10) | 1.04 (0.98-1.10) |

Model 1: adjusted for age, sex, waist circumferences, diabetes family history, alcohol drinking, smoking, regular exercise.

Model 2: adjusted for model 1 plus baseline SBP, LDL-c, log_10_TG, prediabetes, antihypertensive medications, lipid-lowering medications, and mean FPG at 3 visits.

Model 3: adjusted for model 2 plus ΔHOMA-IR.

VVV, visit-to-visit variability; OR, odds ratios; CI, confidence interval; FPG, fasting plasma glucose; ASV, the average successive variability; SBP, systolic blood pressure; LDL-c, low-density lipoprotein cholesterol; log_10_TG, log_10_ transformed triglycerides; HOMA-IR, homeostatic model assessment of insulin resistance index.

Supplementary Table 7. Association of VVV in FPG using VIM with incident diabetes by increased or decreased HOMA-IR between the 2^nd^ and the 3^rd^ visits

| ΔHOMA-IR | Incident cases/  No. of participants | Cumulative  incidence (%) | OR (95% CI) | | |
| --- | --- | --- | --- | --- | --- |
|  |  |  | Model 1 | Model 2 | Model 3 |
| Increased HOMA-IR |  |  |  |  |  |
| Tertiles of VIM (%) |  |  |  |  |  |
| T1 (0-1.26) | 5/295 | 1.7 | 1(ref.) | 1(ref.) | 1(ref.) |
| T2 (1.26-2.02) | 12/340 | 3.5 | 1.96 (0.68-5.65) | 2.13 (0.69-6.55) | 1.93 (0.63-5.91) |
| T3 (2.02-13.08) | 85/444 | 19.1 | 12.92 (5.15-32.39) | 5.43 (2.03-14.53) | 4.13 (1.52-11.24) |
| *P* for trend | / | < 0.001 | < 0.001 | < 0.001 | 0.001 |
| Each 1 increment | / | / | 3.26 (2.62-4.04) | 2.40 (1.83-3.14) | 2.21 (1.67-2.93) |
| Decreased HOMA-IR |  |  |  |  |  |
| Tertiles of VIM (%) |  |  |  |  |  |
| T1 (0-1.26) | 14/327 | 4.3 | 1(ref.) | 1(ref.) | 1(ref.) |
| T2 (1.26-2.02) | 16/275 | 5.8 | 1.28 (0.61-2.69) | 0.88 (0.39-2.02) | 0.88 (0.38-2.01) |
| T3 (2.02-13.08) | 21/175 | 12.0 | 2.81 (1.38-5.72) | 1.46 (0.64-3.31) | 1.46 (0.64-3.30) |
| *P* for trend | / | 0.003 | 0.005 | 0.370 | 0.369 |
| Each 1 increment | / | / | 1.87 (1.44-2.43) | 1.42 (1.02-2.00) | 1.42 (1.02-1.99) |

Model 1: adjusted for age, sex, waist circumferences, diabetes family history, alcohol drinking, smoking, regular exercise.

Model 2: adjusted for model 1 plus baseline SBP, LDL-c, log_10_TG, prediabetes, antihypertensive medications, lipid-lowering medications, and mean FPG at 3 visits.

Model 3: adjusted for model 2 plus ΔHOMA-IR.

VVV, visit-to-visit variability; OR, odds ratios; CI, confidence interval; FPG, fasting plasma glucose; VIM, the variability independent of the mean; SBP, systolic blood pressure; LDL-c, low-density lipoprotein cholesterol; log_10_TG, log_10_ transformed triglycerides; HOMA-IR, homeostatic model assessment of insulin resistance index.

Supplementary Table 8. Odds ratios and 95% confidence intervals of diabetes risks in association with ΔHOMA-IR

| Variable | Incident cases/  No. of participants | Cumulative  incidence (%) | OR (95% CI) | | |
| --- | --- | --- | --- | --- | --- |
|  |  |  | Model 1 | Model 2 | Model 3 |
| ΔHOMA-IR | 153/1856 | 8.2 | 1.70 (1.42-2.02) | 1.64 (1.34-2.01) | 1.81 (1.46-2.25) |

Model 1: adjusted for age, sex, waist circumferences, diabetes family history, alcohol drinking, smoking, regular exercise.

Model 2: adjusted for model 1 plus baseline SBP, LDL-c, log_10_TG, prediabetes, antihypertensive medications, lipid-lowering medications, and mean FPG at 3 visits.

Model 3: adjusted for model 2 plus HOMA-IR at the 2^nd^ visit.

OR, odds ratios; CI, confidence interval; HOMA-IR, homeostatic model assessment of insulin resistance index; SBP, systolic blood pressure; LDL-c, low-density lipoprotein cholesterol; log_10_TG, log_10_ transformed triglycerides; FPG, fasting plasma glucose.
